# Weighted correlation network bioinformatics uncovers a key molecular biosignature driving the left-sided heart failure

**DOI:** 10.1186/s12920-020-00750-9

**Published:** 2020-07-03

**Authors:** Jiamin Zhou, Wei Zhang, Chunying Wei, Zhiliang Zhang, Dasong Yi, Xiaoping Peng, Jingtian Peng, Ran Yin, Zeqi Zheng, Hongmei Qi, Yunfeng Wei, Tong Wen

**Affiliations:** 1 Department of Cardiology, The First Affiliated Hospital of Nanchang University, No. 17 Yongwaizheng Street, Nanchang, 330006 Jiangxi province China; 2 Hypertension Research Institute of Jiangxi Province, Nanchang, 330006 China; 3 Department of Respiratory Medicine, The First Affiliated Hospital of Nanchang University, Nanchang, 330006 China

**Keywords:** Weighted gene co-expression network analysis, Ischemic heart disease, Dilated cardiomyopathy, Heart failure

## Abstract

**Background:**

Left-sided heart failure (HF) is documented as a key prognostic factor in HF. However, the relative molecular mechanisms underlying left-sided HF is unknown. The purpose of this study is to unearth significant modules, pivotal genes and candidate regulatory components governing the progression of left-sided HF by bioinformatical analysis.

**Methods:**

A total of 319 samples in GSE57345 dataset were used for weighted gene correlation network analysis (WGCNA). ClusterProfiler package in R was used to conduct functional enrichment for genes uncovered from the modules of interest. Regulatory networks of genes were built using Cytoscape while Enrichr database was used for identification of transcription factors (TFs). The MCODE plugin was used for identifying hub genes in the modules of interest and their validation was performed based on GSE1869 dataset.

**Results:**

A total of six significant modules were identified. Notably, the blue module was confirmed as the most crucially associated with left-sided HF, ischemic heart disease (ISCH) and dilated cardiomyopathy (CMP). Functional enrichment conveyed that genes belonging to this module were mainly those driving the extracellular matrix-associated processes such as extracellular matrix structural constituent and collagen binding. A total of seven transcriptional factors, including Suppressor of Zeste 12 Protein Homolog (SUZ12) and nuclear factor erythroid 2 like 2 (NFE2L2), adrenergic receptor (AR), were identified as possible regulators of coexpression genes identified in the blue module. A total of three key genes (OGN, HTRA1 and MXRA5) were retained after validation of their prognostic value in left-sided HF. The results of functional enrichment confirmed that these key genes were primarily involved in response to transforming growth factor beta and extracellular matrix.

**Conclusion:**

We uncovered a candidate gene signature correlated with HF, ISCH and CMP in the left ventricle, which may help provide better prognosis and therapeutic decisions and in HF, ISCH and CMP patients.

## Background

Cardiac arrest, the inability of the heart to perform its pumping function, is a major cause of death and a public health problem [1, 2]. The incidence of cardiac arrest is growing worldwide, especially in the vast majority of developed countries [3]. Left-sided heart failure (HF), also known as left ventricular failure, is the common element associated with heart disorders leading to eventual final heart failure [4]. Greater knowledge of the mechanisms involved in the physio-pathogenesis of the left ventricular failure could allow early identification of patients at risk and timely management, which could reduce the socio-economic damages associated with cardiac arrest.

Advances in molecular biology and especially the advent of latest generation sequencing platforms have allowed the accumulation of a large amount of data on the expression of genes regulating the initiation and development of various diseases [5–8]. Correct analysis of these data could help us to uncover the underlying biological functions of genes in different diseases [9, 10]. Regarding cardiac arrest, a number of biomarkers have been discovered in previous studies [11–13]. For example, myoglobin [12], creatine kinase MB isoenzyme (CK-MB) [14], and troponins [15] have been used as biomarkers to assess myocardial pain and diagnose postoperative myocardial infarction. Through transcriptomic analysis, thousands of genes, screened by differential gene expression analysis, have also been suggested as biological markers for cardiac arrest [16]. However, our knowledge on biomarkers especially associated with left ventricular failure is limited. Previous studies based on differential expression analysis have allowed the discovery of key genes between different presentations leading to HF [17, 18], but their application requires experimental validation. However, before any experimental validation, it is essential to do a preliminary work of accurate selection of all the genes very potentially associated with cardiac arrest. This is possible thanks to bioinformatics approaches which are available today.

The WGCNA approach is a bioinformatics technique that allows the extraction and grouping into modules of a list of genes involved in a given biological process [19]. This technique has been used for the credible discovery of a number of genes associated with various diseases and their sub-characteristics [20–23]. WGCNA allows the correlative identification of genes with a similar expression profile to a given characteristic trait. WGCNA has been used to screen genes for different processes in cardiovascular disease such as coronary artery disease [24], congestive HF (CHF) and valvular heart disease (VHD). The use of WGCNA for the discovery of biomarkers related to cardiac arrest after acute myocardial infarction (AMI) has also been reported in a previous study [25] which identified six key genes with a great prognostic value for the progression of HF post-AMI. However, more studies are needed to dissect and decipher the genes involved in left ventricular failure.

Thus, in the present study, we conducted a WGCNA data mining in order to uncover key genes potentially involved in the pathogenesis and the progression of left ventricular failure. Our ultimate goal is to make available a list of biomarkers that could guide the identification of patients at risk of left ventricular failure and the design of appropriate management strategies.

## Methods

### Data sources and data preprocessing

All of the expression datasets used in the present study were obtained from the Gene Expression Omnibus (GEO) datasets (https://www.ncbi.nlm.nih.gov/gds). The data for WGCNA construction was the GSE57345 dataset containing 319 samples of 96 patients with ischemic heart disease (ISCH) and 84 patients with dilated cardiomyopathy (CMP) [26]. The platform used for the acquisition of this data was GPL11532 platform. The validation data was the GSE1869 dataset based on the GPL96 platform and containing samples from six non-HF patients and patients with HF after AMI [27]. The R library “affy” was employed for expression data preprocessing with the Robust Multichip Average (RMA) in the R 3.3.1 software. Following the correction of background effect, quantile normalization and log2-transformation, the datasets were used for subsequent analyses [28].

### Construction of coexpression modules

The WGCNA mining of gene modules was achieved with the R library WGCNA [29]. The standardized connectivity (Z.K) approach was used for identifying outliers suggested by WGCNA authors, with the threshold Z. K score < − 2 as suggested by the WGCNA authors [30]. The gene co-expression similarity Sxy among genes x and y was calculated using the formula Sxy = |cor(x, y)|. The correlation of genes was estimated as follows: axy = |Sxy|β. By using the power gradient method, scale independence as well as mean connectivity were subsequently analyzed. An adequate β value was picked out once the degree of independence was higher than 0.85 [29] for generating a scale-free network. Next, the modules were generated using hierarchical clustering based on average linkage. The module eigengene (ME) was then determined and the module-trait correlations were computed by estimating the correlation among MEs and clinical traits for identifying modules relevant to each clinical trait. The module significance (MS) and the average absolute gene significance (GS) were calculated for evaluating the correlation of overall module genes with the clinical trait. GS is the log10 transformation of *P* values obtained from the linear correlation model based on patient clinical features and gene expression. For the determination of candidate hub genes, the module connectivity (module membership (MM) ≥ median, and gene significance (GS) ≥ median [31]) was applied for selecting the most significant module genes for network construction and visualization in Cytoscape. Next, the MCODE plugin in Cytoscape was used for subnetwork extraction using the following setting: node score cut-off ≥0.2, degree cut-off ≥2, max depth = 100 and K-core ≥2.

### Functional enrichment analysis of genes

The functional enrichment was analyzed by the R library ClusterProfiler [32]. Terms with an enrichment *P* value of < 0.05 were considered as meaningful ones.

### Detection of TFs regulating genes in key module

Module genes were inputted into Enrichr (http://amp.pharm.mssm.edu/Enrichr/) to uncover transcription factors (TFs) interacting with these genes. To reduce false-positives, we screened only TFs with targets available in ENCODE and ChEA gene-set libraries and with corrected *P* value < 0.05 based on the Fisher exact test. The Cytoscape 3.4.0 application (Cytoscape Consortium, SanDiego, CA, USA) was used for TF-target gene network to visualization.

### Validation of hub genes

The GSE1869 data was employed for verification of the hub genes associated with HF. We used the Wilcoxon test to measure the significance of correlation between hub genes’ expression and HF. Additionally, ROC (receiver operating characteristic) analysis was performed on two data sets (GSE57345 and GSE1869) to validate the hub genes, and the area under curve (AUC) of ROC was computed to differentiate HF and non-HF.

## Results

### WGCNA data mining of key modules

**Fig. 1:**
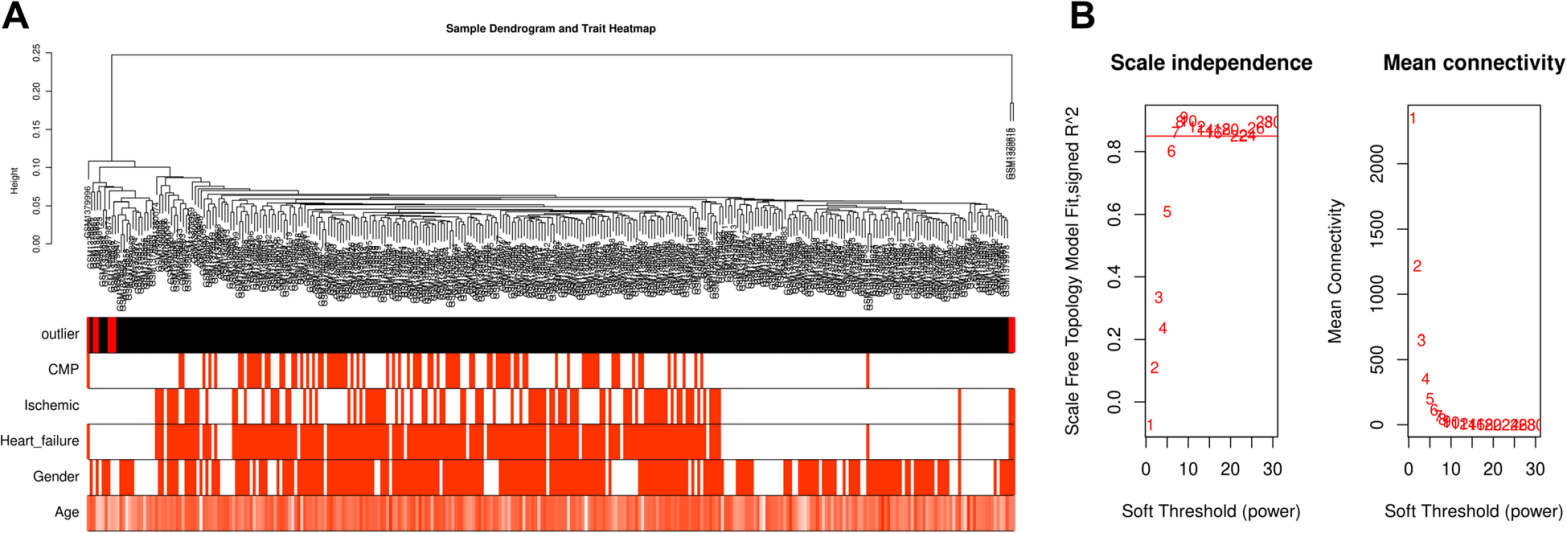
WGCNA based on GSE57345 dataset. **a** Cluster tree and trait heatmap of 319 samples in GSE57345 dataset. **b** Scale-free fit index (left) and average connectivity (right) for determining the threshold powers (β)

**Fig. 2:**
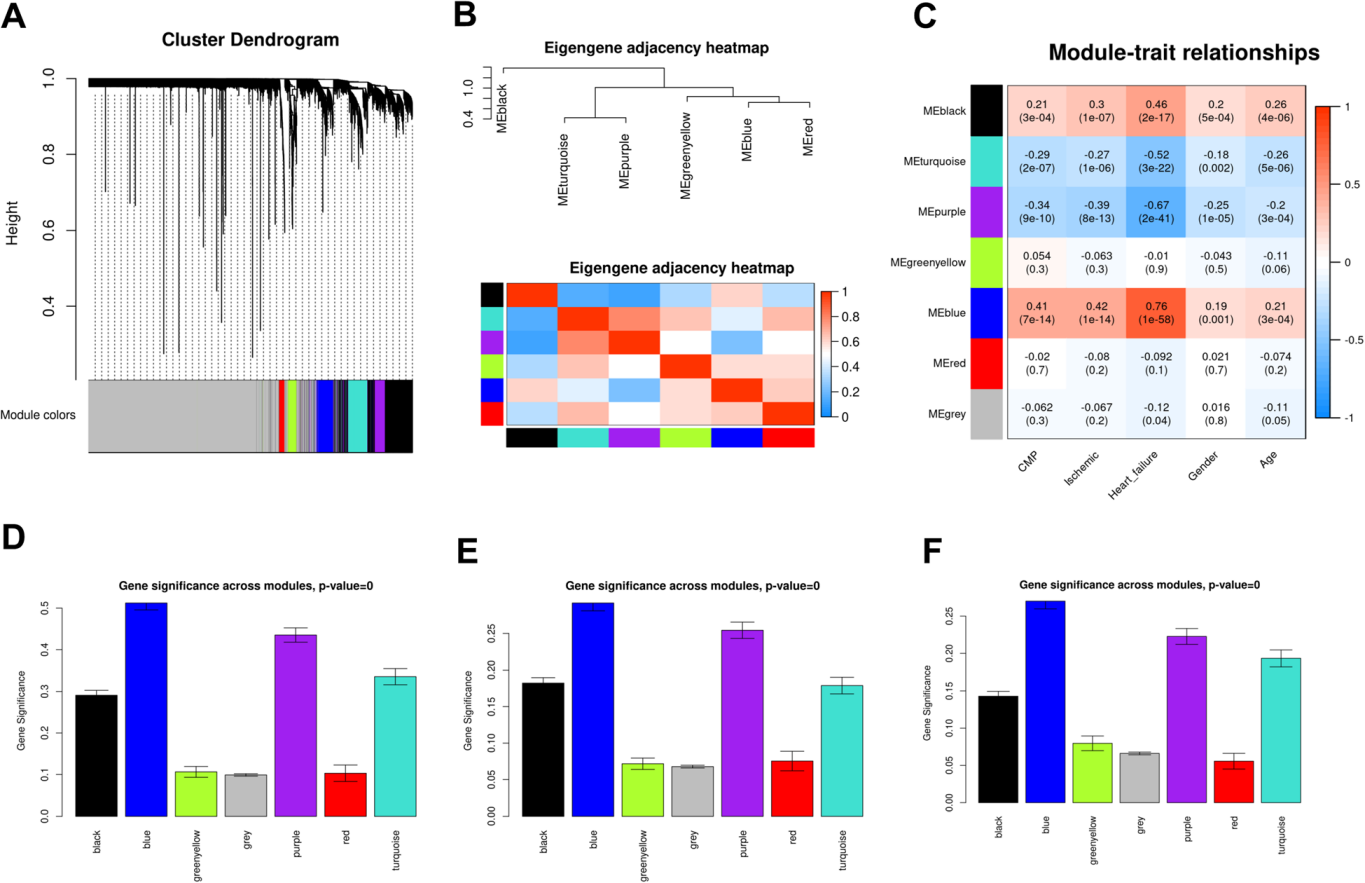
Identification of gene correlation modules. **a** The cluster tree of the common genes in GSE57345 dataset. Each gene was represented by one branch and seven modules were represented by different color in the Figure. **b** Top: Hierarchical clustering dendrogram; bottom: eigengene adjacency heatmap. **c** Module-trait heatmap of correlation amongst the clinical traits of HF and identified modules. **d** Gene significance in the module identified as associated with HF. **e** Gene significance in the module identified as associated with ISCH. **f** Gene significance in the module identified as associated with CMP

Before performing a series of analyses, the GSE57345 dataset was preprocessed. The qualitative assessment of the microarray data was achieved by hierarchical clustering of samples. After detection and elimination of six outliers in the clusters, 313 samples were included in the dendrogram (Fig. 1a). Afterward, β = 7 was chosen as the power value of soft-threshold (Fig. 1b). Then, a total of seven modules were generated by average linkage hierarchical clustering based on the 6230 input genes (Fig. 2a). The Eigengene adjacency heatmap showing the correlation and clustering of the modules was reported in Fig. 2b, and hinted that the six significant correlation modules were divisible into two distinct clusters based on their ME correlation with a great level of independence within the modules. After calculating the MS of each module-trait correlation by WGCNA, the correlation between the available clinical features (HF, ISCH, CMP, Gender and Age) in the GSE57345 dataset with each module was as shown in Fig. 2c. The results showed that the blue module was positively and pointedly associated with the HF, ISCH, and CMP traits (Fig. 2c). In addition, the gene significance in relation with HF, ISCH and CMP across modules were as indicated in Figs. 2d-f, respectively. Since the blue module was the most significantly and positively linked with HF, ISCH, and CMP traits, this module was chosen for further analysis of genes associated with HF, ISCH, and CMP.

### Functions of genes in the blue module

**Fig. 3:**
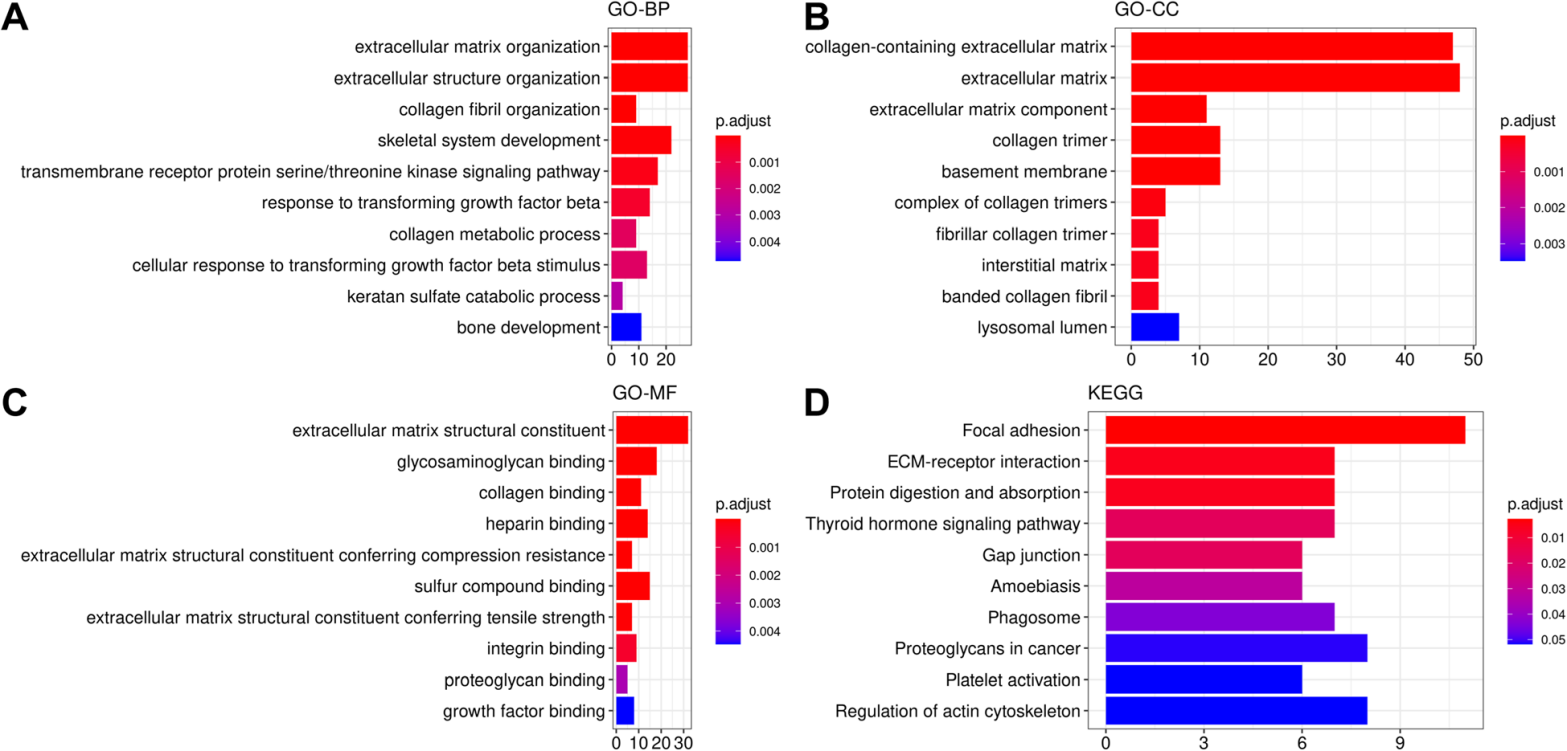
Functional role of genes in the blue module. **a** Terms in Biological Process (GO-BP) obtained from GO enrichment analysis. **b** Terms in Cellular Component (GO-CC) obtained from GO enrichment analysis and **c** Terms in Molecular Function (GO-MF) obtained from GO enrichment analysis. **d** Pathways obtained from KEGG pathway enrichment analysis

The clusterProfiler library was run to uncover the biological meaning of the totality of genes identified in the blue module. These genes were majorly enriched in biological processes (GO-BP) associated with extracellular matrix (Fig. 3a). The most enriched cellular components (GO-CC) were collagen-containing extracellular matrix, extracellular matrix component and extracellular matrix (Fig. 3b) while the most representative molecular functions (GO-MF) were extracellular matrix structural constituent, collagen binding and glycosaminoglycan binding (Fig. 3c). In the KEGG pathway analysis, Focal adhesion and Protein digestion were the overrepresented pathways (Fig. 3d).

### Identification and functional role of hub genes associated with HF

**Fig. 4:**
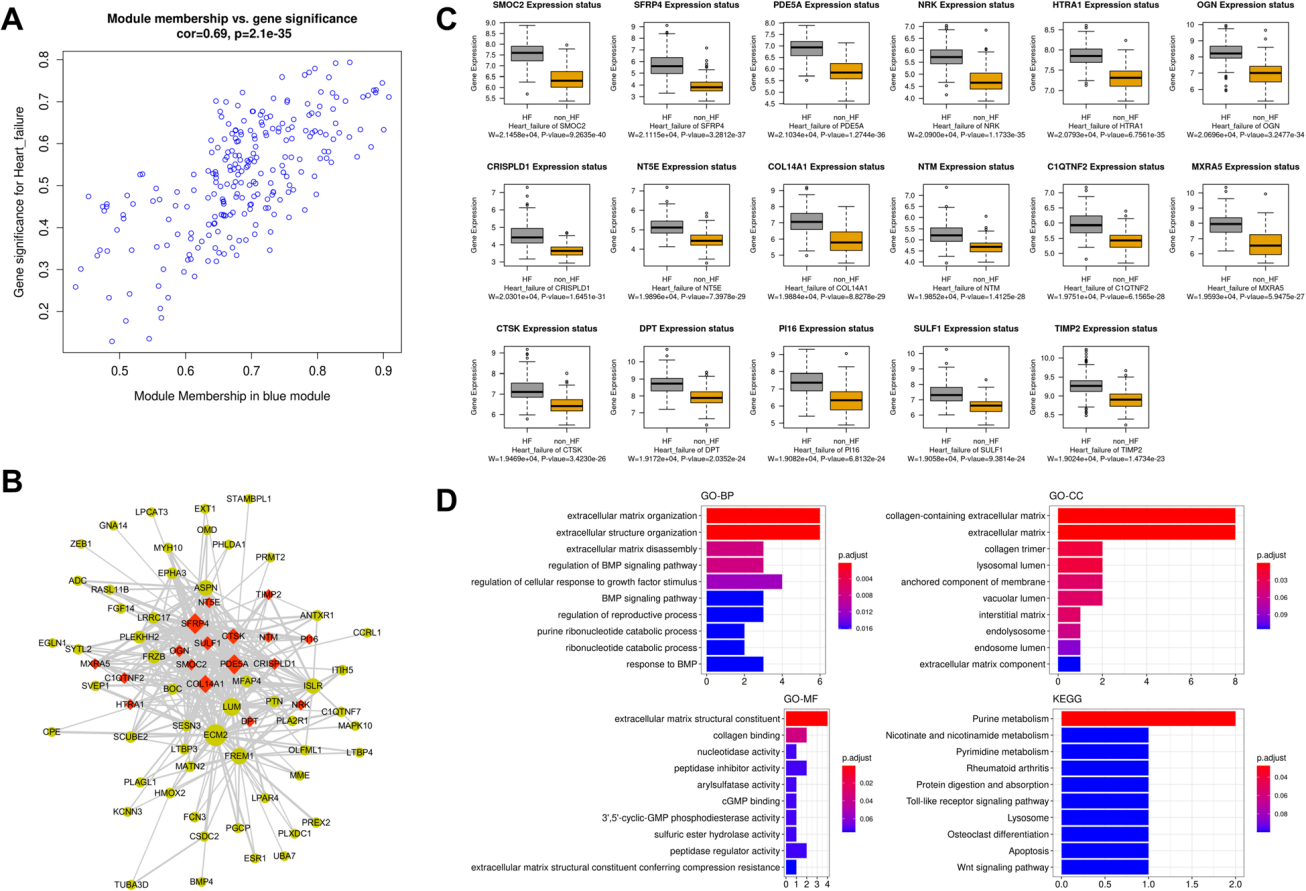
Analysis of the blue module associated with HF. **a** Scatter plots of module membership vs. gene significance for HF. **b** Co-expression regulation network based on genes identified in the blue module. Genes colored in red are hub genes in this network. **c** Boxplots showing the differential expression of hub genes among HF and non-HF specimens. **d** Functional role of hub genes driving HF

The module membership vs. gene significance plot was as depicted in Fig. 4a. The MCODE plugin was used for uncovering the hub genes associated with HF in the blue module from the constructed network. We identified 17 hub genes (TIMP2, SMOC2, NRK, NTM, PDE5A, CTSK, DPT, MXRA5, CRISPLD1, COL14A1, SFRP4, SULF1, OGN, PI16, HTRA1, NT5E, and C1QTNF2) which were colored in red in the network (Fig. 4b). The boxplot showed that these hub genes were significantly upregulated in HF (Fig. 4c). After that, we determined the functional role of hub genes associated with HF in the blue module and found that these genes were those driving the biological processes related to the organization and disassembly of the extracellular matrix (Fig. 4d). In the cellular component ontology, the hub genes were markedly enriched in collagen trimer and extracellular matrix (Fig. 4d). The GO term of collagen binding was the most considerably enriched molecular function (Fig. 4d). Purine metabolism, Nicotinate and nicotinamide metabolism, and Pyrimidine metabolism were the most mainly enriched pathways resulting from the KEGG pathway analysis (Fig. 4d).

### Identification and functional role of hub genes associated with ISCH

**Fig. 5:**
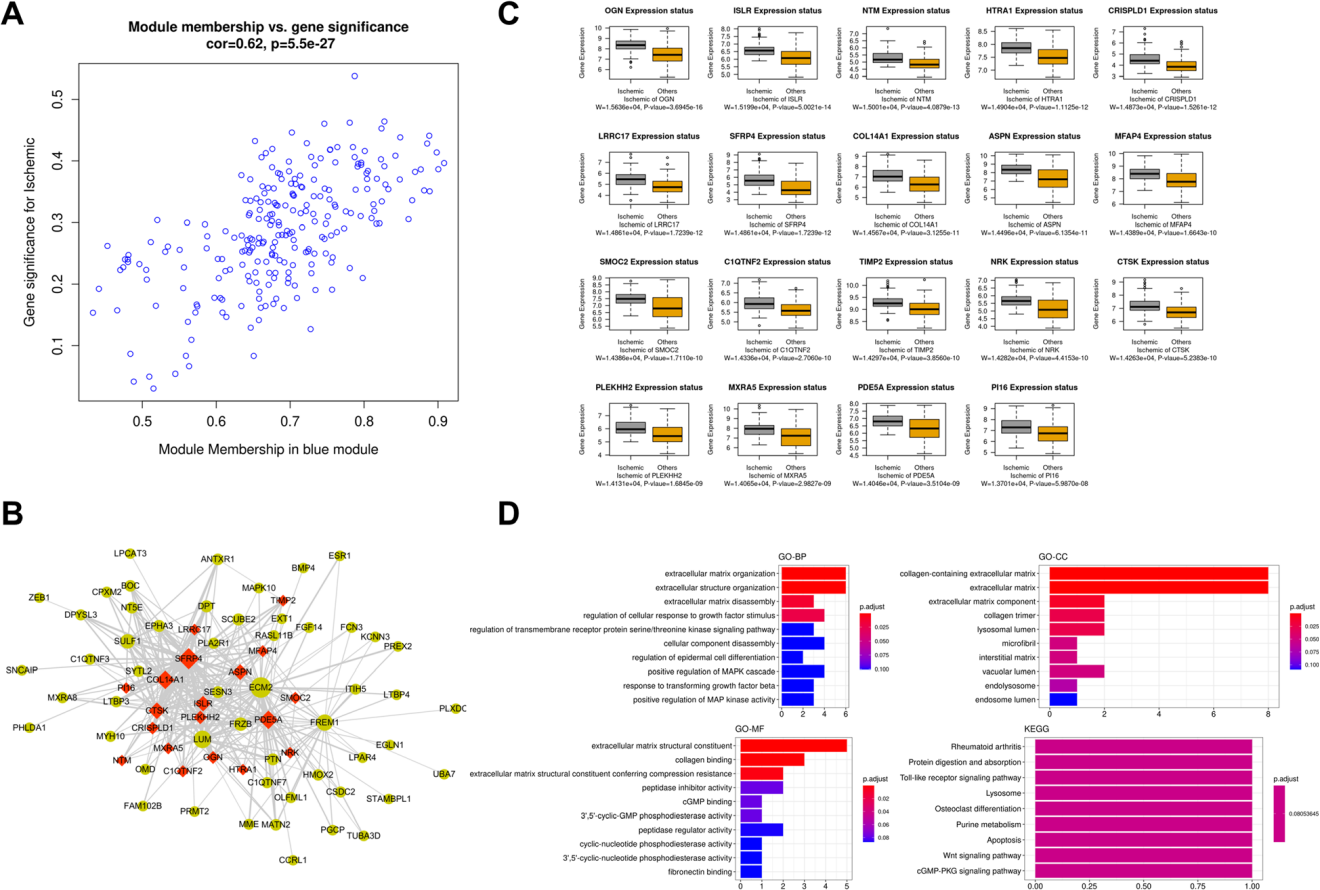
Analysis of the blue module associated with ISCH. **a** Scatter plots of module membership vs. gene significance for ISCH. **b** Co-expression regulation network based on genes identified in the blue module. Genes colored in red are hub genes in this network. **c** Boxplots showing the differential expression of hub genes among ISCH and non-ISCH specimens. **d** Functional role of hub genes driving ISCH

The module membership vs. gene significance plot was as depicted in Fig. 5a. The MCODE plugin was used for uncovering the hub genes related to ISCH in the blue module from the constructed network. We identified 19 hub genes (NTM, ASPN, LRRC17, ISLR, TIMP2, SMOC2, PLEKHH2, NRK, CTSK, PDE5A, MXRA5, CRISPLD1, COL14A1, SFRP4, MFAP4, OGN, PI16, HTRA1, and C1QTNF2) which were colored in red in the network (Fig. 5b). The boxplot of the expression of these key genes showed that all these genes were significantly upregulated in ISCH (Fig. 5c). The analysis of the functions of the hub genes in the blue module associated with ISCH indicated that these genes were those mainly controlling the processes related to extracellular matrix (Fig. 5d). In the cellular component ontology, the hub genes were greatly enriched in collagen-containing extracellular matrix (Fig. 5d). The GO terms of extracellular matrix structural constituent as well as the collagen binding were those significantly enriched molecular functions (Fig. 5d). Rheumatoid arthritis and Protein digestion and absorption were the most significantly enriched pathways resulting from the KEGG pathway analysis (Fig. 5d).

### Identification and functional role of hub genes associated with CMP

**Fig. 6:**
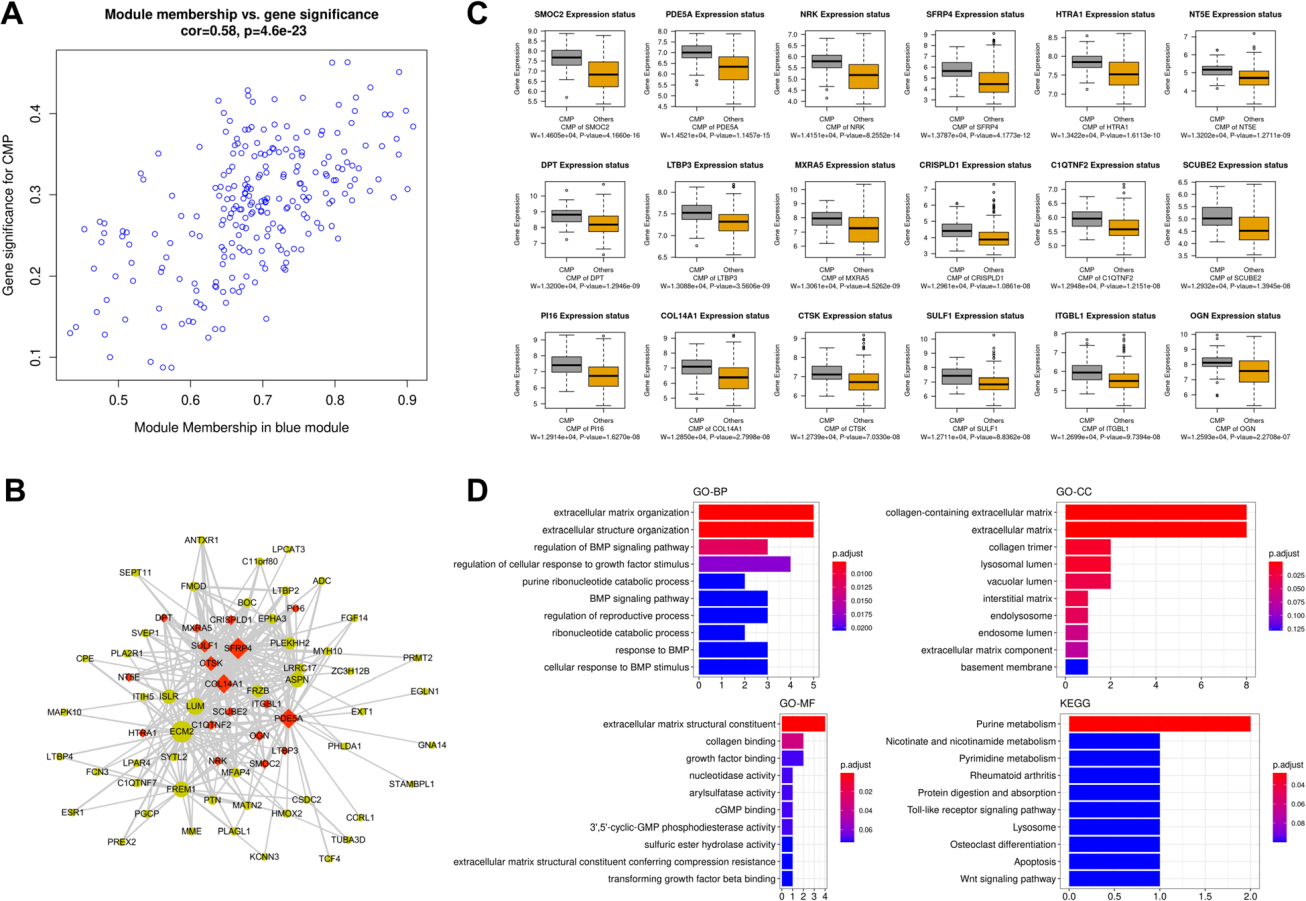
Analysis of the blue module associated with CMP. **a** Scatter plots of module membership vs. gene significance for CMP. **b** Co-expression regulation network based on genes identified in the blue module. Genes colored in red are hub genes in this network. **c** Boxplots showing the differential expression of hub genes among CMP and non-CMP specimens. **d** Functional role of hub genes driving CMP

The module membership vs. gene significance plot was as depicted in Fig. 6a. The MCODE plugin was used for uncovering the hub genes associated with CMP in the blue module from the constructed network. We identified 18 hub genes (SCUBE2, CTSK, ITGBL1, MXRA5, CRISPLD1, SULF1, COL14A1, HTRA1, NT5E, OGN, PI16, C1QTNF2, SMOC2, SFRP4, LTBP3, NRK, PDE5A, and DPT) which were colored in red in the network (Fig. 6b). The boxplot signposted that these hub genes were significantly upregulated in CMP (Fig. 6c). The functional analysis of the hub genes in the blue module associated with CMP indicated their involvement in the biological processes related to extracellular matrix and regulation of BMP signaling pathway (Fig. 6d). In the cellular component ontology, the hub genes were mostly associated with terms related to extracellular matrix and collagen trimer (Fig. 6d). The most enriched GO terms of molecular functions included collagen binding and growth factor binding (Fig. 6d). The result of the KEGG pathway analysis indicated the involvement of hub genes in Purine metabolism, Nicotinate and nicotinamide metabolism, and Pyrimidine metabolism pathways (Fig. 6d).

### Identification of transcription factors associated with genes in blue module

**Fig. 7:**
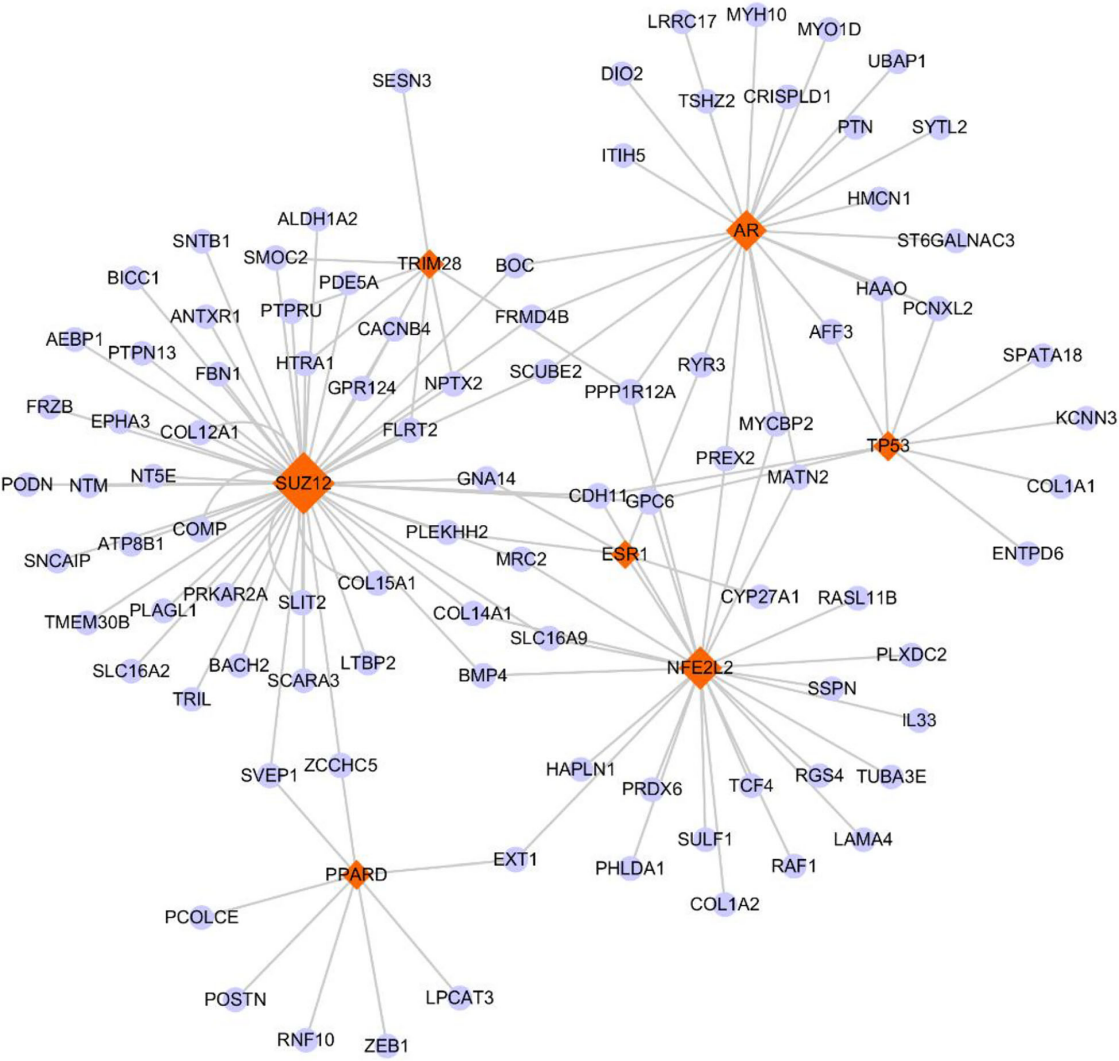
Gene-transcription factor network in the blue module. Red diamonds and blue nodes represent the transcription factors and genes, respectively

In order to explore transcription factors (TFs) controlling gene expression in the blue module, we analyzed ENCODE and ChEA which were the data sources available in Enrich. By setting an adjusted *P*-value cutoff of 0.05, a total of seven TFs were revealed (Additional file 1). The most prevalent TFs were SUZ12 with 47 target genes, NFE2L2 with 26 target genes and AR with 23 target genes. The regulatory network of TF-target gene based on all the genes in blue module and the seven TFs was as displayed in Fig. 7.

### Validation of hub genes

**Fig. 8:**
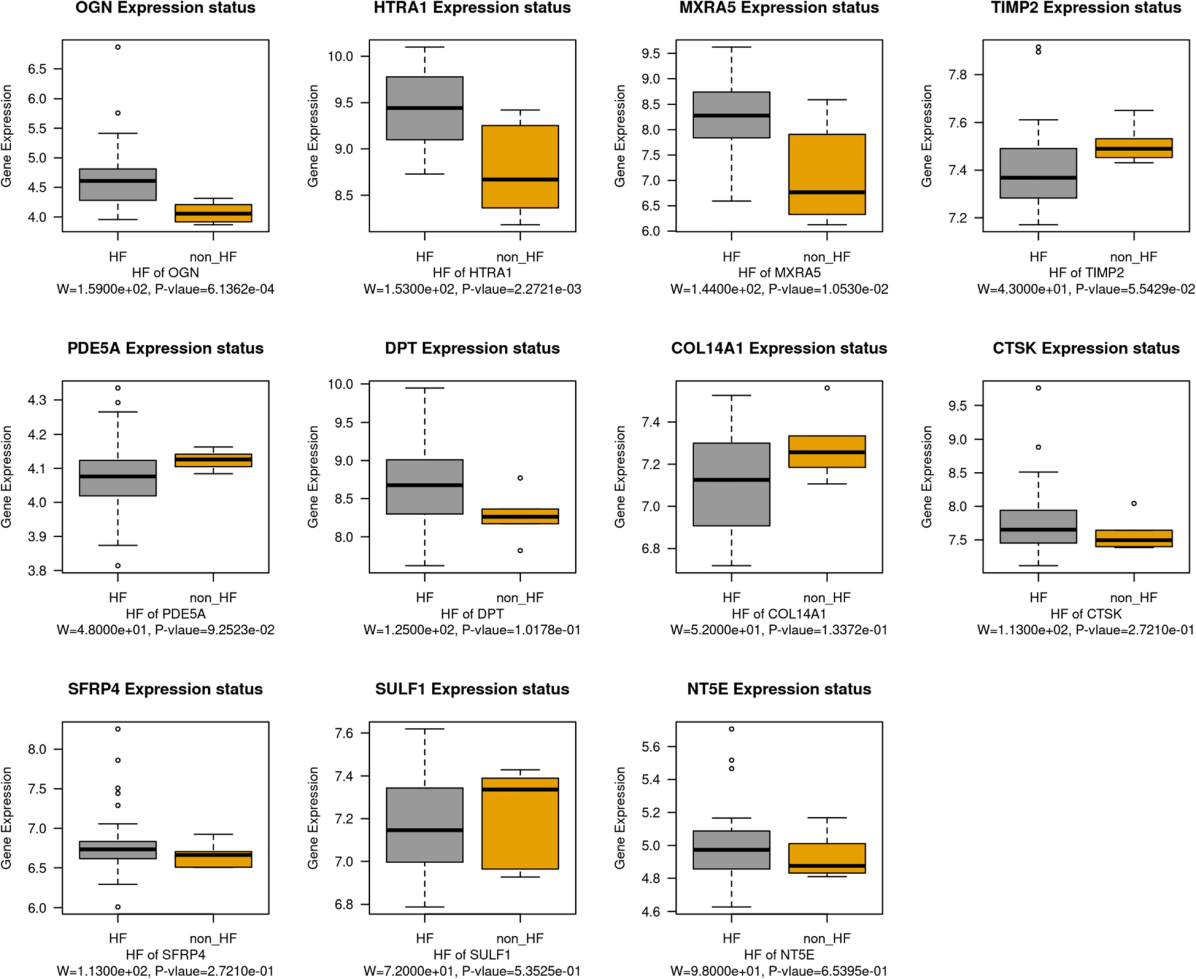
Differential expression of hub genes between HF and non-HF specimens in the validation dataset GSE1869

**Fig. 9:**
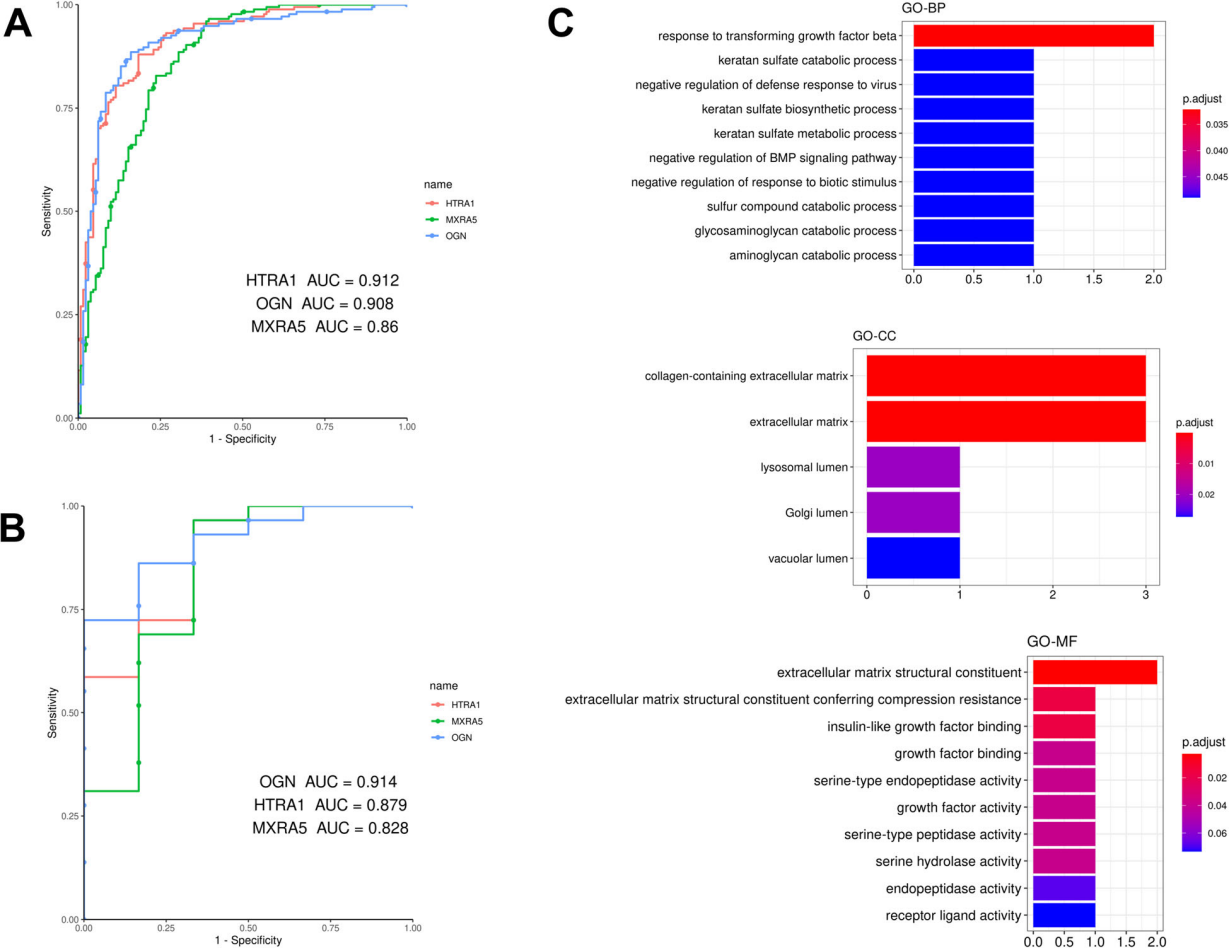
Analysis of the key genes (HTRA1, OGN and MXRA5). ROC curve analysis of the key genes in (**a**) GSE57345 and (**b**) GSE1869 datasets; (**c**) Functional annotation for the key genes, which included GO analysis and KEGG pathway enrichment analysis

All the hub genes of HF were selected for validation by using GSE1869 data set. However, only 11 hub genes were obtained in the GSE1869, which included OGN, HTRA1, MXRA5, TIMP2, PDE5A, DPT, COL14A1, CTSK, SFRP4, SULF1, and NT5E. The differential expression of hub genes between HF and non-HF samples showed that the overexpression of OGN, HTRA1 and MXRA5 were closely related to the occurrence of HF (Fig. 8). Thus, we speculated that such three genes were the key genes of HF progression. To validate the hub genes as predictive biomarkers of HF, we performed and calculated ROC curves [33] and the AUCs [95% confidence intervals (CIs)], respectively. The AUCs of OGN, HTRA1 and MXRA5 in the GSE57345 were respectively 0.912, 0.908 and 0.878, suggesting OGN, HTRA1 and MXRA5 as potential biomarkers of HF (Fig. 9a). The AUCs of OGN, HTRA1 and MXRA5 in the GSE1869 were respectively 0.914, 0.879 and 0.828, further indicating OGN, HTRA1 and MXRA5 as potential biomarkers of HF (Fig. 9b). We also found the AUC of each validated gene was higher than 0.7, indicating that OGN, HTRA1 and MXRA5 could effectively distinguish HF and non-HF. Therefore, such genes were selected as the true key genes associated with HF. Further functional annotation of these true key genes was performed, and the result showed that these genes were majorly implicated in response to transforming growth factor beta, extracellular matrix and extracellular matrix structural constituent (Fig. 9c).

## Discussion

The most frequent subtypes of HF include ischemic heart disease (ISCH) and dilated cardiomyopathy (CMP). ISCH is due to the shrinkage of blood supply to the myocardium, while the heart of CMP becomes weakened and enlarged [34]. ISCH and CMP are conductive to symptoms similar to HF, but accumulating findings suggested that these two subtypes might respond differently to therapy [35, 36]. The new era of high-throughput technologies has experienced tremendous progress in the development of computational algorithms for bioinformatics purposes [37, 38]. In this current study, we employed the WGCNA data mining approach to identify genes that are significantly altered upon HF. Then we discovered the crucial modules markedly related to HF development, ISCH and CMP. Finally, the practicality of the key genes as prognostic biomarkers for HF was also evaluated.

In the WGCNA, we screened six significant gene modules from the GSE57345 dataset. The blue module was uncovered as the most crucially correlated with the status of HF, ISCH and CMP in patients, thus we chose the blue module as the main module for the subsequent analysis. Our study hinted that genes clustered in the correlation gene expression module were chiefly implicated in regulation of ECM (extracellular matrix), including ECM organization and ECM receptor interaction. The ECM network plays an important role in cardiac homeostasis, not only by providing structural support, but also by transducing key signals to cardiomyocytes, vascular cells, and interstitial cells [39]. It is known that the alterations of ECM homeostasis may lead to diastolic or systolic dysfunction in heart and consequent development of HF [40]. A study conducted by Tsoutsman revealed that modulation of CCN2 on early ECM changes might provide a new therapeutic target in the treatment of HF [41].

After identifying the hub genes in the blue module underlying the studied traits (HF, ISCH and CMP), we identified 12 hub genes as those common to the three studied traits including SMOC2, NRK, PDE5A, CTSK, MXRA5, CRISPLD1, COL14A1, SFRP4, OGN, PI16, HTRA1 and C1QTNF2. Next, we found the functional enrichment of the hub genes associated with HF or ISCH or CMP were similar with those for the blue module. Williams and his colleagues found that SMOC2 was differentially expressed in failing right ventricular, and was potential targets for further study on HF [42]. A study revealed that SMOC2 could modulate fibroblast proliferation and extracellular matrix deposition [43]. Similarly, a study revealed that expression of C1QTNF2 related to anti-fibrotic function [44]. Thus, SMOC2 and C1QTNF2 might play a similar function to protect from cardiac fibrosis. Multiple studies have reported the pathophysiological role of cyclic guanosine monophosphate (cGMP) signaling in HF. Increased levels of cGMP have been demonstrated to exhibit cardioprotective effects in many cardiovascular diseases. PDE5A is a leading factor contributing to cGMP signaling and cardiac hypertrophy. Multiple studies suggested that PDE5A inhibitor could effectively limit myocardial injury caused by stresses [45–47]. As a lysosomal cysteine protease, CTSK has been intensively investigated in the osteoporosis [48–50]. In recent years, reports hinted that activation of lysosomal cysteine protease might exert a deleterious role in the progression of cardiometabolic diseases [51]. Researchers have conveyed that CTSK may become an alternative therapeutic target for cardiac disease [52]. In a latest study, CRISPLD1 has been suggested to used be as a novel conserved target in the management of HF [53]. MXRA5 is a cancer related gene and several studies indicated the potential value of this gene as a novel therapeutic target for various cancers including colorectal cancer [54], non-small cell lung cancer [55] and glioblastoma multiform [56]. There are few reports about function of MXRA5 in HF. Studies have shown that SFRP4 is expressed in cardiomyocytes, and elevated during HF. Similarly to our results, studies demonstrated that OGN is significantly up-regulated in CMP and ISCH by reducing cardiac inflammation and injury, and, thus, could become a promising biomarker for HF [57]. The roles of NRK, COL14A1, PI16 and HTRA1 in HF have not been investigated in the past. It is worth noting that most of these common genes have been proven as genes associated with the regulation of ECM, which corroborated with the functional annotation of the hub genes. Therefore, these genes (SMOC2, NRK, PDE5A, CTSK, MXRA5, CRISPLD1, COL14A1, SFRP4, OGN, PI16, HTRA1 and C1QTNF2) might be the biomarkers of HF, which could regulate organization of ECM to affect HF progression. Our present findings hinted that these genes could be crucial for understanding the pathogenesis of HF and even constitute relevant therapeutic targets.

As regulators of gene expression, TFs are closely linked with the pathogenesis of various diseases. Herein, we explored the possible regulation of genes in the blue module by TFs, and identified seven TFs, which included SUZ12, NFE2L2, TRIM28, AR, TP53, PPAR and ESR1. A new report revealed that SUZ12 (Suppressor of Zeste 12 Protein Homolog) could mediate the downregulation of myocyte enhancer factor 2A, thereby preventing cardiac hypertrophy [58]. It is well known that NFE2L2 (nuclear factor erythroid 2 like 2) is a critical TF that can induce adaptive responses against oxidative stress (OS) for maintaining cellular redox balance [59]. Accumulating researches demonstrated that the upregulation of NFE2L2 could protect the myocardium from ischemic injury and may be of therapeutic benefit in the treatment of ISCH [60]. AR (adrenergic receptor) overactivation is reported as a factor involved in the pathogenesis of HF [61]. Taken together, our findings indicated that these TFs formed a compact regulatory network with genes uncovered from the blue module, and the changes of the activities of these TFs may play crucial functions in the initiation and progression of HF, ISCH and CMP.

In addition, we verified the hub genes associated with HF based on the GSE1869 data set. However, among these 17 hub genes, only OGN, HTRA1, MXRA5, TIMP2, PDE5A, DPT, COL14A1, CTSK, SFRP4, SULF1, and NT5E were obtained in this data set. Then we used Wilcoxon test to determine whether these genes were significantly linked with HF status. A total of three genes were significantly correlated with HF status (*P* < 0.05), which included OGN, HTRA1 and MXRA5. We further validated their value for HF progression and prognosis by performing ROC curve analysis. In many researches, the ROC curve was applied to assess the performance of diagnosis, and the area under the ROC curve (AUC) is a commonly used summary index for comparison among multiple ROC curves [62–64]. In this study, the ROC analysis proved that the selected three key genes could be used as potential biomarkers with great specificity and sensitivity for prognosis in HF patients.

## Conclusions

The current study applied the WGCNA data mining and other bioinformatics approaches to identify and validate the major module and corresponding hub genes involved in the progression of HF, ISCH and CMP. The blue module was identified as the common key module among three studied traits. Eleven hub genes, namely SMOC2, NRK, PDE5A, CTSK, MXRA5, CRISPLD1, COL14A1, SFRP4, OGN, PI16, HTRA1 and C1QTNF2 are likely to be prognostic biomarkers for development of HF. Afterward, TFs (SUZ12, NFE2L2, TRIM28, AR, TP53, PPAR and ESR1) were predicted as key regulators that contribute to the pathophysiological outcomes of HF. Finally, we screened three key genes (OGN, HTRA1 and MXRA5) which showed great specificity and sensitivity for HF prognosis in further validation. Though this study is a preliminary investigation, our findings proposed new potential therapeutic targets of HF, and provide novel insights in the pathogenesis of HF.

## Supplementary information

**Additional file 1.** ENCODE analysis of transcription factors.

## Data Availability

The datasets, GSE57345 (https://www.ncbi.nlm.nih.gov/geo/query/acc.cgi?acc=GSE57345) and GSE1869 (https://www.ncbi.nlm.nih.gov/geo/query/acc.cgi?acc=GSE1869), analyzed during the current study are available in the Gene Expression Omnibus (GEO) datasets at the National Center for Biotechnology Information (https://www.ncbi.nlm.nih.gov/gds).

## Abbreviations

CK-MB: Creatine kinase MB isoenzyme
CHF: Congestive heart failure
VHD: Valvular heart disease
CMP: Dilated cardiomyopathy
GO: Gene ontology
TF: Transcription factor
ROC: Receiver operating characteristic
ISCH: Ischemic heart disease
ECM: Extracellular matrix
cGMP: Cyclic guanosine monophosphate
CTSK: Cathepsin K
OS: Oxidative stress
OGN: Osteoglycin
